# Immune‐related adverse events and outcomes among pan‐cancer patients receiving immune checkpoint inhibitors: A monocentric real‐world observational study

**DOI:** 10.1002/cam4.6449

**Published:** 2023-08-11

**Authors:** Xiaoxiao Ge, Weiping Jiang, Hongqing Li, Yanxu Wu, Xiangyang Li, Shaohua Cui

**Affiliations:** ^1^ Department of Pulmonary and Critical Care Medicine Huadong Hospital, Fudan University Shanghai China

**Keywords:** immune‐related adverse events, immunotherapy, solid tumor, survival

## Abstract

**Background:**

Real‐world evidence on immune‐related adverse events (irAEs) are relatively insufficient. Herein patterns and outcomes of irAEs after administration of anti‐programmed cell death 1 (PD‐1) and its legend 1 (PD‐L1) antibodies were investigated.

**Methods:**

Patients treated with anti‐PD‐1/PD‐L1 drugs from January 2018 to September 2021 at Huadong Hospital, Fudan University were included. Common Terminology Criteria for Adverse Events (CTCAE) was used for irAEs evaluation. The primary endpoints were the clinical description of irAEs.

**Results:**

Two hundred and forty‐one solid tumor patients were included, with lung cancer as the most common tumor type (56%). 187 (77.6%) patients presented any kind of irAEs. The median time to any irAE onset was 28 (95% CI 24–32) days. Skin toxicities are the most common irAEs (46.1%) and the irAEs (36.5%) occurred earliest after immune‐checkpoint inhibitors. The most frequently occurred all‐grade irAEs were rash (23.7%), myelosuppression (20.7%), and hepatic injury (19.5%). 23 (9.5%) patients died of severe irAEs, which consists of 10 patients with pneumonitis, four colitis, four myocarditis, and one each for gastritis, pulmonary embolism, myelosuppression, hypophysitis, and encephalitis. Patients with any irAE onset had significantly longer progression‐free survival (PFS) (*p* = 0.013) and overall survival (OS) (*p* = 0.007), respectively, than patients without irAEs. In addition, patients with skin toxicities (*p* = 0.012) or blood toxicities (*p* = 0.015) had achieved a longer PFS, than those without corresponding toxitities, respectively.

**Conclusion:**

Most irAEs are mild and manageable, while some irAEs can present at later time or can be life‐threatening, especially pneumonitis as we observed. Patients with any irAE onset may achieve a better prognosis than those without irAEs, and presentation of skin or blood toxicities will indicate a better PFS.

## 1 INTRODUCTION

Immune‐checkpoint inhibitors (ICIs) have emerged as a milestone of cancer therapy.^1^ Programmed cell death 1 (PD‐1) and its ligand 1 (PD‐L1) are key molecules mediating immune tolerance, thus monoclonal antibodies blocking this pathway can enhance immune systems to fight against tumors.^2^, ^3^, ^4^, ^5^ Clinically, except for monotherapy, ICIs are also being used in combination regimens, including those involving chemotherapy, targeted drugs, or other types of ICIs.^6^ Despite durable responses in both advanced and perioperative settings, administrations of ICIs can lead to a broad spectrum of adverse events termed the immune‐related adverse events (irAEs).^7^, ^8^

IrAEs represent a great challenge in clinical practice due to its universality and complexity. Firstly, irAEs can affect any organ system, especially organs with extensive environmental interfaces or harboring presumed pre‐existing autoimmunity.^7^, ^8^ Secondly, the severity of irAEs can range from asymptomatic to life‐threatening,^9^ and fatal irAEs are commonly occurred early after treatment.^10^ IrAEs can occur during therapy or even several months after ICI cessation, although they most often present early after ICI treatment.^11^ Interestingly, the occurrence of irAEs may predict patients' prognosis.^12^, ^13^, ^14^ Therefore, monitoring and timely handling irAEs are of critical importance. Albeit most mild‐to‐moderate irAEs can be well managed by clinical observation and symptomatic treatment without withholding the ICIs, discontinuation of ICIs and medical interventions are still needed for patients who present severe irAEs.^15^, ^16^, ^17^

Currently, most knowledge of our understanding of irAEs comes from clinical trials, which have rigorous inclusion and exclusion criteria. Charateristics of irAEs, such as incidence, patterns of time to onset, and time to recovery in real‐world settings, are relatively unknown. In this real‐world observational study, we investigated the patterns and outcomes of irAEs presented in a pan‐cancer cohort treated with PD‐1/PD‐L1 inhibitors. Relationships between irAEs onset and patients' progression‐free survival (PFS) and overall survival (OS) were also studied.

## 2 PATIENTS AND METHODS

### 2.1 Study population

**FIGURE 1:**
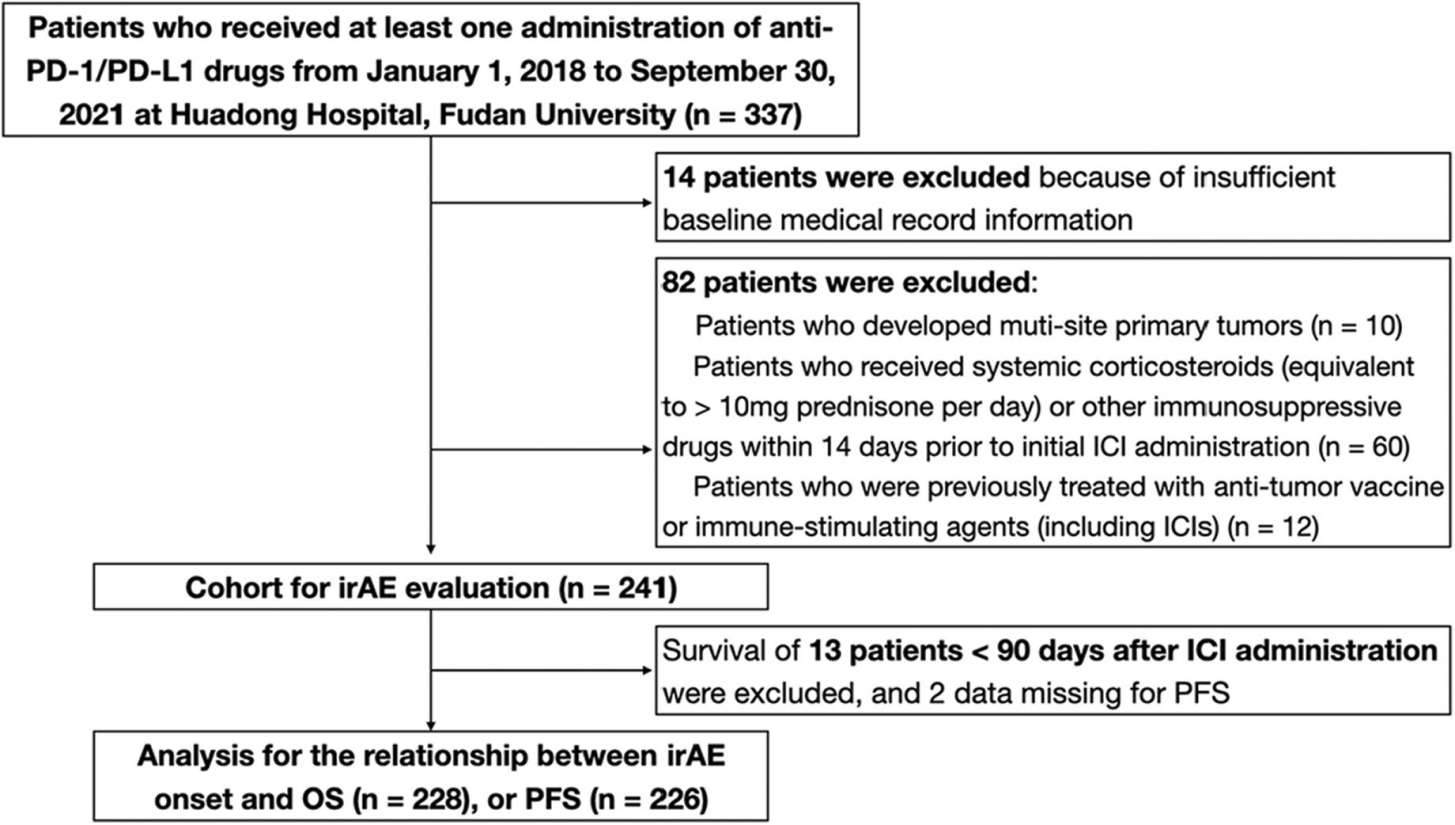
Patient flow chart. IrAE, immune‐related adverse event; ICI, immune checkpoint inhibitor; OS, overall survival; PD‐1, programmed cell death 1; PD‐L1, programmed cell death ligand 1; PFS, progression‐free survival.

A monocentric real‐world study was conducted at Huadong Hospital, Fudan University. Patients who received at least one administration of anti‐PD‐1/PD‐L1 drugs from January 1, 2018 to September 30, 2021 were included. Patients eligible for inclusion were those who were histologically diagnosed with non‐metastatic or metastatic solid tumors, aged ≥18 years (except for lymphoma which may develop at earlier ages), treated with ICIs as a single agent or in combination with other standard antitumor strategies such as chemotherapy. ICIs used in the perioperative period or for advanced stages were both allowed. Exclusion criteria were patients who developed multi‐site primary tumors, received systemic corticosteroids (equivalent to >10 mg prednisone per day), or other immunosuppressive drugs within 14 days prior to initial ICI administration, previously treated with antitumor vaccine or immune‐stimulating agents (including ICIs), or those with insufficient information (Figure 1).

This study was approved by the Ethics Committee of Huadong Hospital, Fudan University, Shanghai (Ethical approval number: 20220100). This study was performed in accordance with the Declaration of Helsinki.

### 2.2 Data collection and adverse events registration

Data regarding patient demography and clinical characteristics were collected from electronic medical records. Tumor responses were assessed using the Response Evaluation Criteria in Solid Tumors (RECIST), version 1.1. All irAEs were recorded and classified based on the affected organ. In addition, subtypes of irAEs were classified according to the organ/system and the degree of affectation according to the Common Terminology Criteria for Adverse Events (CTCAE) of the National Cancer Institute (version 5.01).

All irAEs were analyzed, according to medical notes and our follow‐up data by phone and/or outpatient service. Two reviewers (Ge X and Cui S) evaluated the irAEs based on medical records and will have a discussion with all authors if inconsistences occur. In order to homogeneously record the adverse events, all were classified according to the CTCAE (version 5.01), such as gastrointestinal disorders, respiratory disorders, skin and subcutaneous disorders, and endocrine disorders, and etc. Additionally, each adverse event is graded as Grade 1 to Grade 4 (or Grade 5) according to CTCAE criteria. Of note, myelosuppression is recorded as one type of irAE in this study as we would like to provide information on the occurrence of myelosuppression after the use of ICIs combined with chemotherapy in real‐world settings, considering most patients in our study cohort received a combination regimen containing an ICI plus chemotherapy. Cutoff date of this study was September 30, 2022.

### 2.3 Outcomes and definitions

The primary endpoints were the clinical descriptions of irAEs after anti‐PD‐1/PD‐L1 treatment. Secondary endpoints were PFS and OS descriptions, and the correlations between irAEs onset and PFS or OS. Time to onset was defined as the time between the date ICI was first administered and the date of first clinical, radiographic, or biological abnormalities of irAEs. Time to recovery was defined as the date from the initiation of irAEs to the date when irAEs were resolved. PFS was calculated as the time from the date ICIs were first administered until the date of objective progressive disease (PD) (or the date of recurrence for patients who received radical operation) according to RECIST, or all‐cause death. OS was defined as the time between the date ICIs were first administered until date of death from any reason.

### 2.4 Statistical analysis

IrAEs were evaluated in the whole cohort. Incidence rate, grade, median onset time, clinical management, proportions of recovery with median recovery time, proportions of irAEs leading to ICIs discontinuation, dose reduction, delayed prescription, or death were analyzed. PFS and OS was evaluated in the population whose survival were more than 90 days after ICIs treatment. Any irAE, specific irAEs with an incidence of ≥10% were included in survival analysis. For the correlation of any irAE onset and PFS or OS, analysis was also done in the whole cohort as a sensitivity evaluation.

Categorical variables were described with the use of frequency counts and percentages, while continuous variables were described with the use of mean or median and range. The Kaplan–Meier method was applied to estimate time‐to‐event variables. For PFS and OS, a Cox regression model with *Enter* method was used to estimate hazard ratios (HRs), and to compare differences between groups. Adjusted *p* values and HRs were given after adjustment for gender, age, smoking status, ICI drugs, treatment timing, combinations, and Eastern Cooperative Oncology Group (ECOG) Performance Status (PS). *p* values less than 0.05 were considered statistically significant. Statistical analysis was performed with IBM SPSS Statistics (IBM, version 24, Armonk, NY, USA). Density plots and boxplots were generated using R (version 4.2.0).

## 3 RESULTS

### 3.1 Patients characteristics

A total of 241 patients received at least one administration of anti‐PD‐1 or anti‐PD‐L1 inhibitors at our center were enrolled (Figure 1). Demographic and clinicopathologic characteristics of the cohort are shown in Table 1 (Specific ICIs used for the cohort are shown in Table S1). The mean age of the cohort was 60.9 (range 14–86) years. Most patients were males (74.7%) and never smokers (52.3%). The most common tumor type was lung cancer (56%), followed by nasopharyngeal carcinoma (11.6%), esophagus carcinoma (10.8%), and liver cancer (6.2%). 216 (89.6%) patients were at advanced stage prior to ICIs treatment. Additionally, 61 (25.3%) patients received ICI monotherapy while 180 (74.7%) were prescribed with combination therapies, with chemotherapy (63.5%; 153/241) as the most common combinations.

**TABLE 1:** Demographic and clinicopathologic characteristics of the 241 patients.

Characteristics		*N* (%)
Age, years	Mean (Range)	60.9 (14–86)
Age, years	< 65	131 (54.4)
	≥ 65	110 (45.6)
Gender	Male	180 (74.7)
	Female	61 (25.3)
Smoking status	Never smokers	126 (52.3)
	Ever/current smokers	115 (47.7)
PD‐L1 TPS	No expression/Negative	54 (22.4)
	< 1%	8 (3.3)
	1% ~ 49%	33 (13.7)
	≥ 50%	29 (12.0)
	Unknown	117 (48.5)
Tumor type^a^	Lung cancer	135 (56.0)
	Nasopharyngeal carcinoma	28 (11.6)
	Esophagus carcinoma	26 (10.8)
	Liver cancer	15 (6.2)
	Gastrointestinal cancer	8 (3.3)
	Others	29 (12.0)
Subtypes of lung cancer^a^	Adenocarcinoma	80 (33.2)
	Squamous cell carcinoma	33 (13.7)
	Small cell carcinoma	13 (5.4)
	Others	9 (3.7)
Driver genes^a^	EGFR	14 (5.8)
	ROS1	1 (0.4)
	Negative	108 (44.8)
	Unknown	12 (5.0)
Stage Prior to ICIs	Early stage	25 (10.4)
	Advanced stage	216 (89.6)
Timing of ICIs	Perioperative	21 (8.7)
	First line	110 (45.6)
	Second line	71 (29.5)
	Third or later lines	39 (16.2)
Combinations with ICIs	No combinations	61 (25.3)
	Chemotherapy	153 (63.5)
	Targeted therapy	8 (3.3)
	VEGF inhibitors	17 (7.1)
	Others	2 (0.8)
ICI drugs	Anti‐PD‐1 drugs	229 (95.0)
	Anti‐PD‐L1 drugs	12 (5.0)
ECOG PS	≤ 1	210 (87.1)
	≥ 2	31 (12.9)

Abbreviations: ECOG PS, Eastern Cooperative Oncology Group Performance Status; ICI, immune checkpoint inhibitor; PD‐1, programmed cell death 1; PD‐L1, programmed cell death ligand 1; TPS, Tumor proportion score.

^a^ Only for lung cancer.

### 3.2 General irAEs incidence

General information for irAEs is shown in Table 2. With the median follow‐up time of 455 (95%CI 416–494) days, 187 (77.6%) patients presented any kind of irAE. The median time to any irAE onset (first occurred) was 28 (95% CI 24–32) days. At the date cutoff, 86 (35.7%), 64 (26.6%), and 25 (10.4%) patients suffered from irAEs occurred in 1, 2, and 3 organ/systems, respectively. Among all organ/systems, skin was the most common (36.5%) to be affected first after ICI, followed by endocrine (9.5%), blood (9.5%), and liver (8.7%). Furthermore, other organ/systems such as lung, kidney, nerves, and heart can also serve as the first affected sites by irAEs after ICIs treatment. By the study cutoff date, 142 (58.9%) patients survived. 23 (23.2%; 23/99) patients were died as a result of severe irAEs.

**TABLE 2:** General information for irAEs of the study cohort.

General Information		N (%)
Median follow‐up time		455 (95% CI 416–494) days
Mean time to first irAE onset		55 (95% CI 42.0–68) days
Median time to first irAE onset		28 (95% CI 24–32) days
Patients with any irAE onset		187 (77.6)
Total numbers of irAE onset	0	54 (22.4)
	1	86 (35.7)
	2	64 (26.6)
	3	25 (10.4)
	4	11 (4.6)
	5	1 (0.4)
System/Organs first presented	Skin	88 (36.5)
	Endocrine	23 (9.5)
	Hematological	23 (9.5)
	Liver	21 (8.7)
	GI	11 (4.6)
	Lung	8 (3.3)
	Kidney	4 (1.7)
	Nerves	3 (1.2)
	Heart	2 (0.8)
	Vascular	2 (0.8)
	Rheumatism	1 (0.4)
	Eye	1 (0.4)
Proportion of surviving patients		142 (58.9)
Proportion of patients died because of irAEs		23/99 (23.2)

Abbreviations: CI, confidence interval; GI, gastrointestinal; IrAE, immune‐related adverse event.

### 3.3 IrAEs by specific sites

**FIGURE 2:**
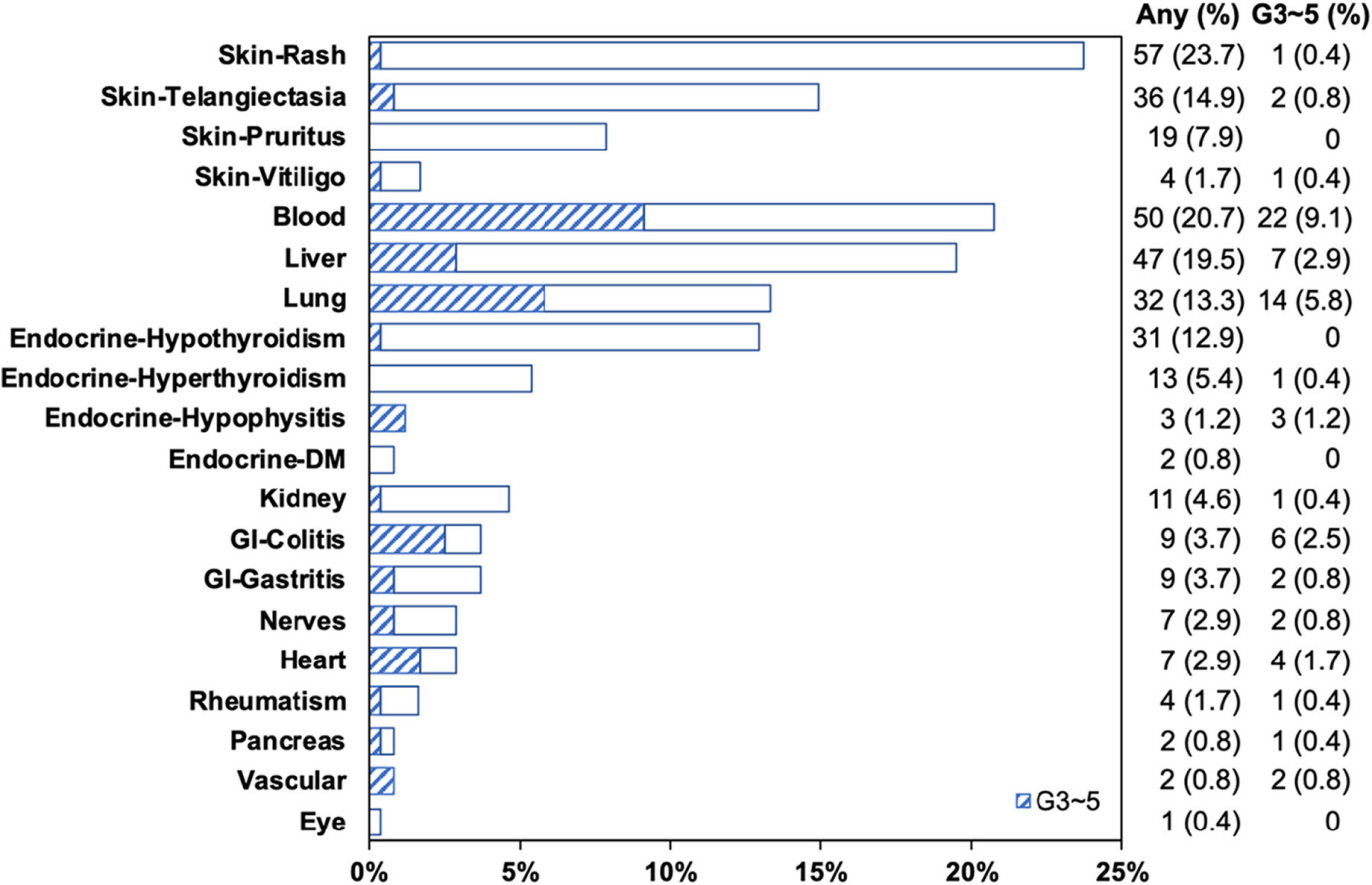
Details of plots showing the percentage of patients who presented specific irAEs (any grade and grades 3 ~ 5). IrAE, immune‐related adverse event; DM, diabetes mellitus; GI, gastrointestinal.

IrAEs stratified by systems/organs are summarized in Table 3 and Figure 2. Skin, endocrine, and GI toxicities contained different manifestations, and proportions of patients presenting at least one of these manifestations were 46.1% (111/241), 17.4% (42/241), and 7.1% (17/241), respectively. The most frequently occurring all‐grade irAEs were rash (23.7%), myelosuppression (20.7%), and hepatic injury (19.5%). Other all‐grade irAEs whose incidence rate exceeded 10% included telangiectasia (14.9%), pneumonitis (13.3%), and hypothyroidism (12.9%). Most irAEs were mild (Grade 1 ~ 2) and could be managed. Grade 3 ~ 5 irAEs exceeding 5% incidence included myelosuppression (9.1%) and pneumonitis (5.8%). 23 (9.5%) patients died of severe irAEs, which consisted of 10 patients with pneumonitis, four with colitis, four with myocarditis, and one each for gastritis, pulmonary embolism, myelosuppression, hypophysitis, and encephalitis.

**TABLE 3:** Incidence, severity, management and outcomes of irAEs stratified by systems/organs.

IrAEs	Grade (N = 241), *n* (%)			Median onset, days (range)	Specific management, n (%)	Recovery, n (%)	Media*n* time to recovery, days (range)	IrAE leading to ICI adjustment or death, *n* (%)			
	Any	1 ~ 2	3 ~ 5					Discontinuation	Delay	Dose reduction	Death
Lung											
Pneumonitis	32 (13.3)	18 (7.5)	14 (5.8)	88 (8–731)	26/32 (81.3)	20/32 (62.5)	145 (39–526)	19/32 (59.4)	3/32 (9.4)	0	10/32 (31.3)
GI											
Gastritis	9 (3.7)	7 (2.9)	2 (0.8)	20 (1–80)	9/9 (100)	8/9 (88.9)	66 (38–366)	1/9 (11.1)	3/9 (33.3)	0	1/9 (11.1)
Colitis	9 (3.7)	3 (1.2)	6 (2.5)	26 (1–277)	9/9 (100)	5/9 (55.6)	76 (5–240)	6/9 (66.7)	1/9 (11.1)	0	4/9 (44.4)
Rheumatism											
Arthritis/ myositis	4 (1.7)	3 (1.2)	1 (0.4)	31 (27–152)	4/4 (100)	3/4 (75)	58 (39–99)	0	0	0	0
Liver											
Hepatic insufficiency	47 (19.5)	40 (16.6)	7 (2.9)	48 (4–751)	47/47 (100)	46/47 (97.9)	60 (6–320)	5/47 (10.6)	11/47 (23.4)	0	0
Kidney											
Renal insufficiency	11 (4.6)	10 (4.2)	1 (0.4)	46 (21–184)	11/11 (100)	6/11 (54.5)	145 (3–194)	5/11 (45.5)	3/11 (27.3)	0	0
Heart											
Myocarditis	7 (2.9)	3 (1.2)	4 (1.7)	69 (21–295)	7/7 (100)	3/7 (42.9)	64 (59–158)	6/7 (85.7)	1/7 (14.3)	1/7 (14.3)	4/7 (57.1)
Vascular											
Pulmonary embolism	2 (0.8)	0	2 (0.8)	4 (4–30)	2 (100)	1/2 (50)	235	1/2 (50)	0	0	1/2 (50)
Eye											
Uveitis	1 (0.4)	1 (0.4)	0	67	1/1 (100)	1/1 (100)	48	0	0	0	0
Pancreas											
Pancreatitis	2 (0.8)	1 (0.4)	1 (0.4)	50 (50–208)	2/2 (100)	2/2 (100)	31 (31–32)	1/2 (50)	0	0	0
Blood											
Myelosuppression	50 (20.7)	28 (11.6)	22 (9.1)	34 (2–477)	50/50 (100)	45/50 (90)	80 (4–575)	7/50 (14)	5/50 (10)	2/50 (4)	1/50 (2)
Endocrine											
Hyperthyroidism	13 (5.4)	13 (5.4)	0	38 (11–418)	1/13 (8)	6/13 (46.2)	46 (39–83)	0	0	0	0
Hypothyroidism	31 (12.9)	30 (12.5)	1 (0.4)	143 (10–677)	24/31 (77.4)	25/31 (80.6)	142 (26–645)	2/31 (6.5)	2/31 (6.5)	0	0
Diabetes mellitus	2 (0.8)	2 (0.8)	0	14 (14–64)	2/2 (100)	2/2 (100)	58 (58–220)	0	0	0	0
Hypophysitis	3 (1.2)	0	3 (1.2)	127 (16–176)	3/3 (100)	2/3 (66.7)	187 (187–476)	3/3 (100)	0	0	1/3 (33.3)
Skin											
Rash	57 (23.7)	56 (23.3)	1 (0.4)	27 (1–90)	51/57 (89.5)	55/57 (96.5)	127 (23–754)	6/57 (10.5)	6/57 (10.5)	0	0
Pruritus	19 (7.9)	19 (7.9)	0	36 (2–400)	15/19 (78.9)	16/19 (84.2)	118 (31–426)	0	2/19 (10.5)	0	0
Vitiligo	4 (1.7)	3 (1.3)	1 (0.4)	24 (14–35)	4/4 (100)	3/4 (75)	128 (86–430)	1/4 (25)	0	0	0
Telangiectasia	36 (14.9)	34 (14.1)	2 (0.8)	34 (13–100)	20/36 (55.6)	30/36 (83.3)	151 (16–747)	6/36 (16.7)	3/36 (8.3)	0	0
Nerves											
Encephalitis/peripheral neuritis	7 (2.9)	5 (2.1)	2 (0.8)	62 (22–513)	7/7 (100)	5/7 (71.4)	156 (57–275)	2/7 (28.6)	1/7 (14.3)	0	1/7 (14.3)

Abbreviations: GI, gastrointestinal; IrAE, immune‐related adverse event.

**FIGURE 3:**
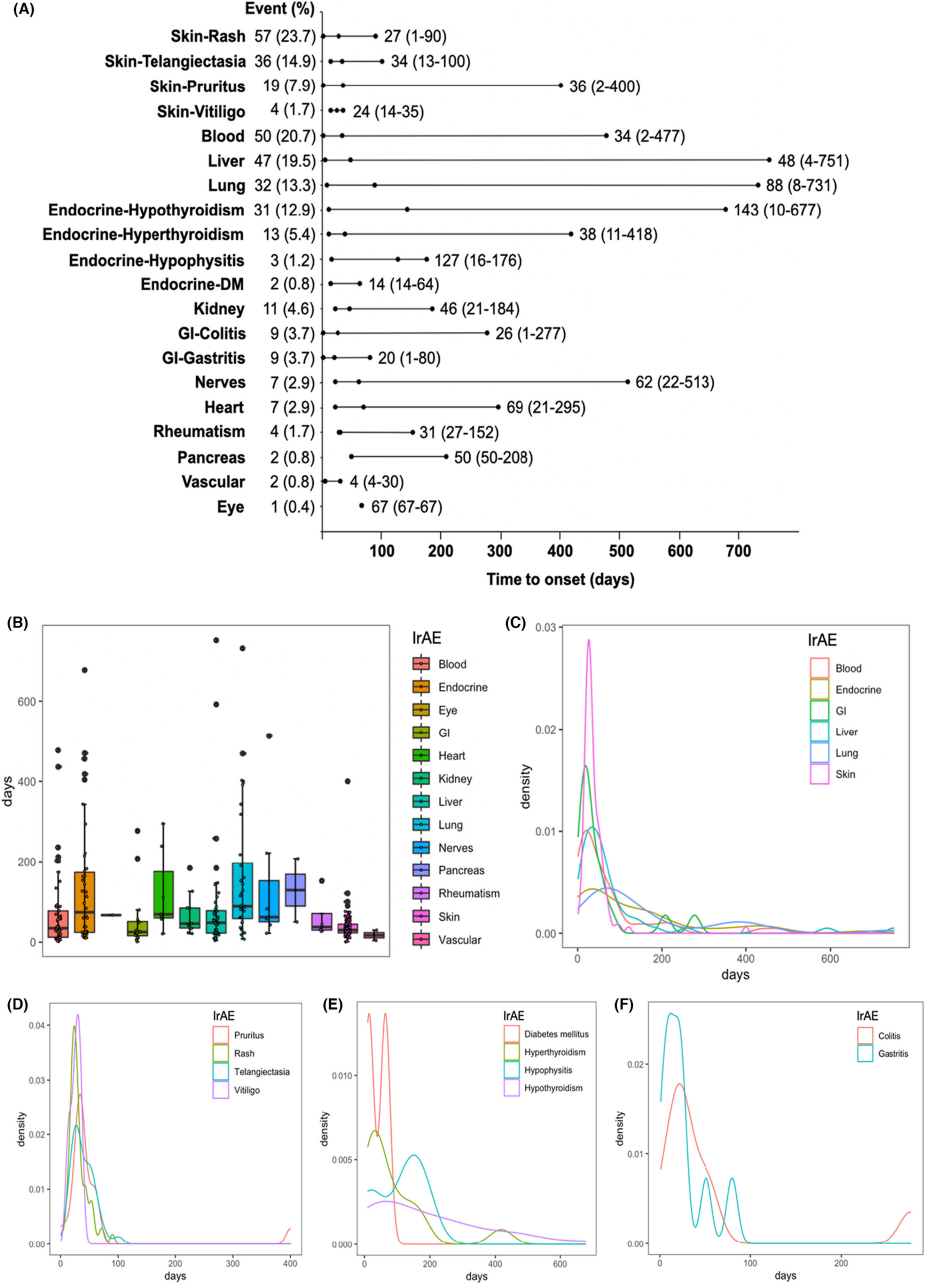
Time to onset of irAEs stratified by systems/organs. (A) Plots showing details of time to onsets for specific irAEs (median and range). (B) Boxplots showing the percentage of patients who presented specific irAEs. (C) Density plots showing time to onset of irAEs with incidence rate ≥5%. (D ~ F) Density plots showing time to onset of blood (D), endocrine (E) and GI (F) toxicities by organs. IrAE, immune‐related adverse event; DM, diabetes mellitus; GI, gastrointestinal.

The time to onset of each irAE is also calculated (Table 3 and Figure 3). In the light of median time to irAEs onset, most irAEs occurred early after ICIs treatment. Pulmonary embolism (4 [range 4–30] days), diabetes mellitus (14 [14–64] days), gastritis (20 [1–80] days), vitiligo (24 [14–35] days), colitis (26 [1–277] days), and rash (27 [1–90] days) occurred within 30 days after initial ICIs treatment. However, nerve toxities (62 [22–513]), myocarditis (69 [21–295] days), pneumonitis (88 [8–731] days), hypophysitis (127 [16–176] days), and hypothyroidism (143 [10–677] days) occurred after 60 days after the initial administration of ICI.

**FIGURE 4:**
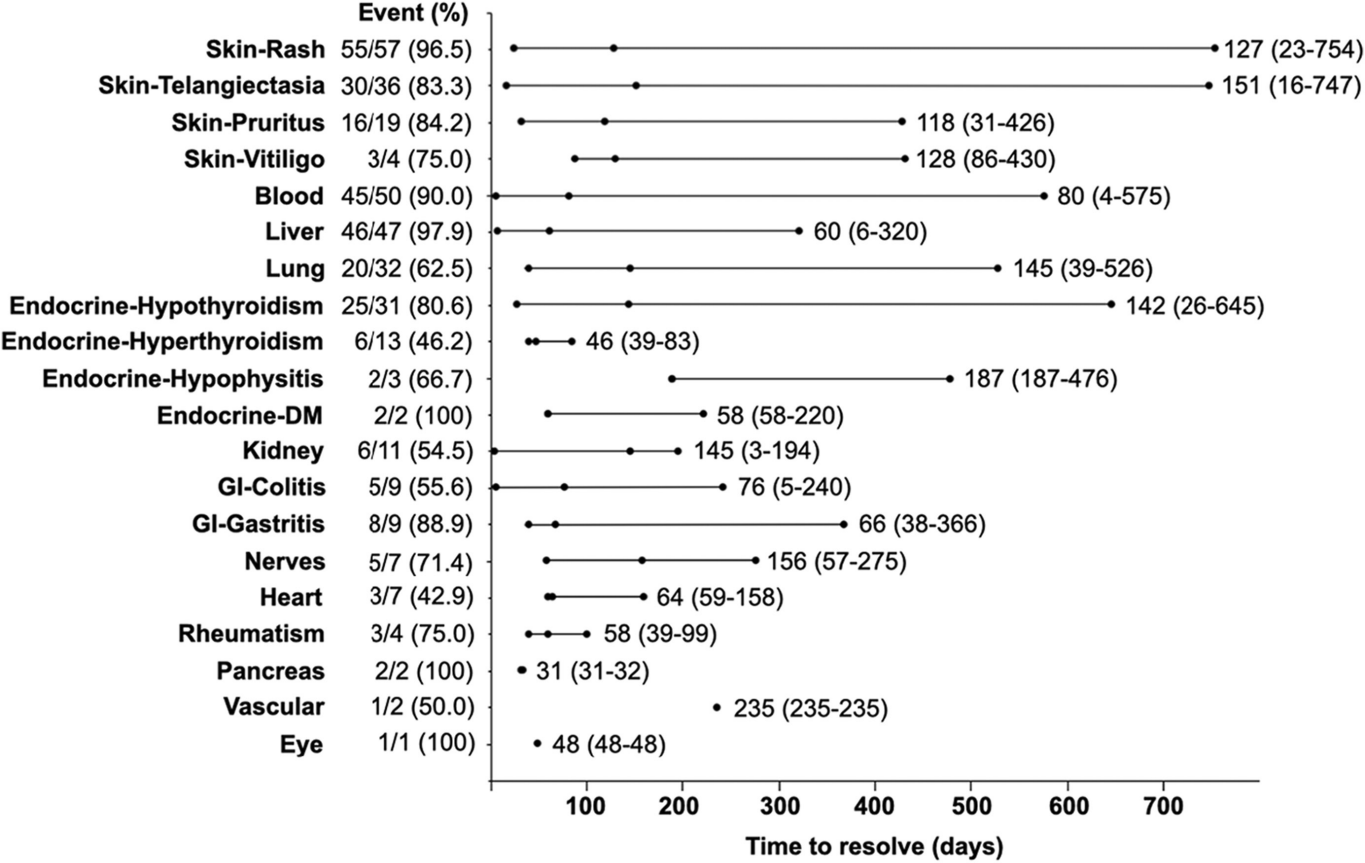
Plots showing details of recovery time for specific irAEs (median and range). IrAE, immune‐related adverse event; DM, diabetes mellitus; GI, gastrointestinal.

In our cohort, most irAEs were managed with symptomatic treatment and finally recovered (Table 3 and Figure 4). The median time to recovery varied among different irAEs. For skin toxicities, the median recovery time was between 118 and 151 days. Median time to recovery for myelosuppression, hepatic injury, pneumonitis, and hypothyroidism were 80 (range 4–575) days, 60 (6–320) days, 145 (39–526) days, and 142 (26–645) days, respectively. In addition, six patients (46.2%; 6/13) with hyperthyroidism transferred to hypothyroidism after a median time of 93 (range 21–393) days.

### 3.4 Relationship of irAEs onset and PFS or OS

**FIGURE 5:**
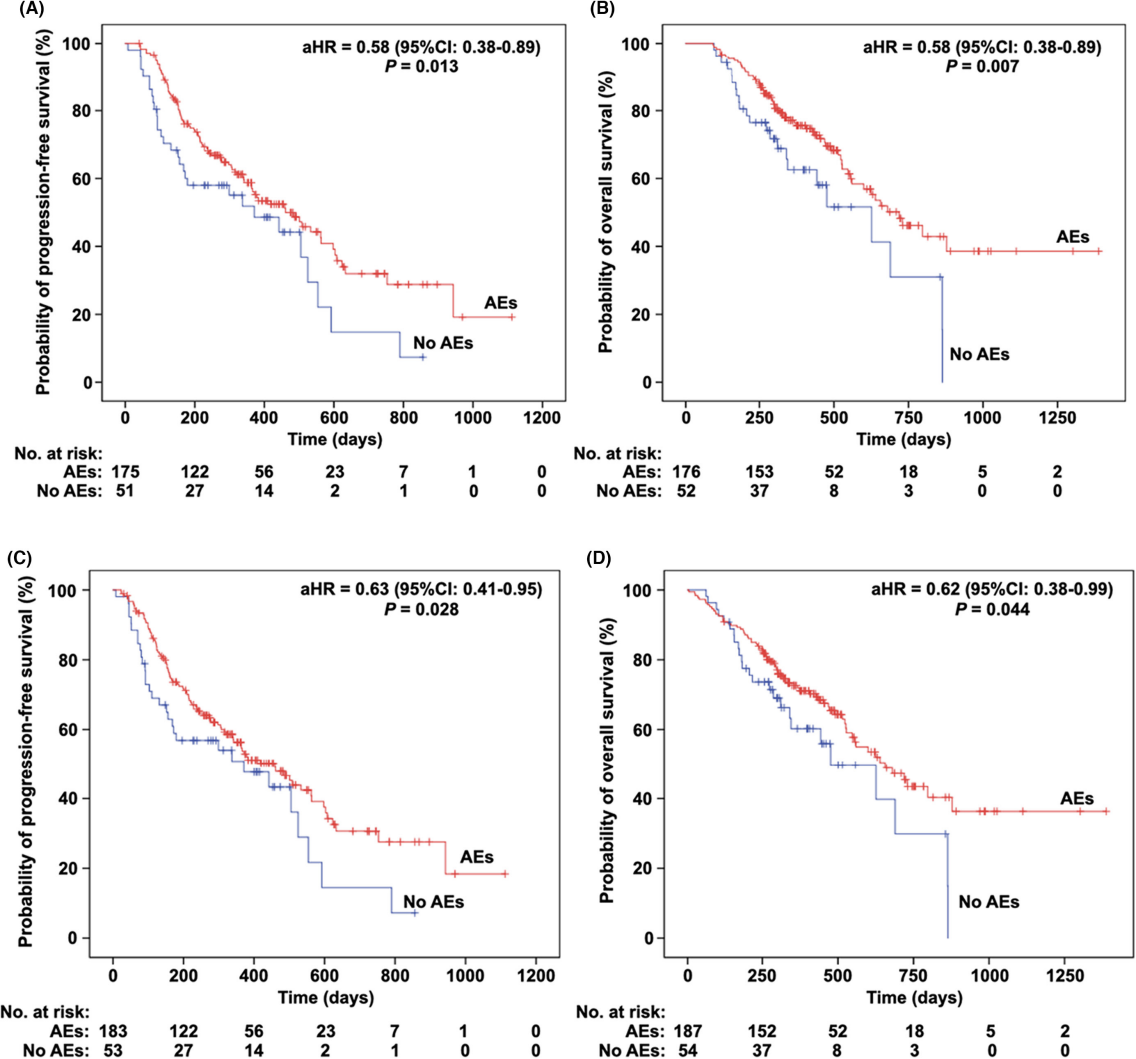
Kaplan–Meier curves of PFS and OS by irAEs onset. (A, B) Main analysis for PFS (A) and OS (B) in the population with a survival time of more than 90 days. (C, D) Sensitivity analysis for PFS (C) and OS (D), which was done in all cohort. *P* and HR values were adjusted for gender, age, smoking status, ICI drugs, treatment timing, combinations, and ECOG PS. Two and six missing data for PFS in main analysis and sensitivity analysis, respectively. aHR, adjusted hazard ratio; ECOG PS, Eastern Cooperative Oncology Group (ECOG) Performance Status; ICI, immune checkpoint inhibitor; PFS, progression‐free survival; OS, overall survival.

At the study cutoff date, 119 (52.7%; 119/226) and 86 (37.7%; 86/228) patients had PFS and OS events, respectively. Median PFS and median OS of the cohort were 459 (95% CI 364–554) and 656 (95% CI 553–759) days, respectively. Patients with any irAE onset had significantly longer PFS (461 [95% CI 336–586] vs. 371 (173–569) days, aHR = 0.58 [95% CI 0.38–0.89], *p* = 0.013) and OS (719 [568–870] vs. 626 (371–881) days, aHR = 0.58 [95% CI 0.38–0.89], *p* = 0.007), respectively, than patients without irAEs (Table 4, Figure 5A,B). Sensitivity analysis achieved consistent results (Figure 5C,D).

**TABLE 4:** PFS and OS for patients presented with irAEs versus patients without irAEs.

IrAEs	PFS (*n* = 226)				OS (*n* = 228)			
	Onset, median (95%CI) days	No irAEs, median (95% CI) days	*P*	aHR (95% CI)	Onset, median (95% CI) days	No irAEs, median (95% CI) days	*P*	aHR (95% CI)
Any	461 (336–586)	371 (173–569)	0.013	0.58 (0.38–0.89)	719 (568–870)	626 (371–881)	0.007	0.58 (0.38–0.89)
Skin	508 (408–608)	371 (248–494)	0.012	0.62 (0.43–0.90)	797 (562–1032)	626 (469–783)	0.055	0.65 (0.42–1.01)
Blood	623	418 (328–508)	0.015	0.73 (0.56–0.94)	730	626 (503–749)	0.188	0.82 (0.61–1.10)
Liver	364 (133–595)	459 (362–556)	0.383	0.89 (0.70–1.15)	719 (383–1055)	656 (512–800)	0.763	0.95 (0.71–1.28)
Endocrine	459 (303–615)	442 (328–556)	0.366	0.89 (0.70–1.14)	NR	639 (478–800)	0.100	0.77 (0.56–1.05)
Lung	379 (50–708)	459 (358–560)	0.679	0.94 (0.71–1.25)	560 (553–759)	689 (589–789)	0.791	0.96 (0.70–1.31)

Abbreviations: aHR, adjusted hazard ratio; CI, confidence interval; IrAE, immune‐related adverse event; NR, not reached; OS, overall survival; PFS, progression‐free survival.

Additionally, longer PFS was achieved in patients with skin toxicities (508 [408–608] vs. 371 [248–494] days, aHR = 0.62 [95% CI 0.43–0.90], *p* = 0.012) and blood toxicities (623 vs. 418 [328–508] days, aHR = 0.73 [0.56–0.94], *p* = 0.015), compared with patients without corresponding toxicities (Table 4).

## 4 DISCUSSION

This study provides real‐world evidence of the characteristics and outcomes of irAEs, after ICI administration in pan‐cancer patients. Data from up to nine types of 241 patients in our center were included.

Presence of irAEs after PD‐1/PD‐L1 inhibitors is common. According to our results and previous experience, the incidence of all‐grade irAEs can be as high as 70%–80%.^18^, ^19^ Although irAEs can occur in any organ/system, skin is the most frequently affected organ and skin toxicities tend to appear within the first three months of ICI treatment.^20^ Consistent with previous findings, skin toxicities were the most common and the earliest to be presented in our cohort. Rash, pruritus, vitiligo, and telangiectasia were the main manifestations of skin toxicities, and most of the irAEs were mild (Grade 1 ~ 2) and reversible.

Other common irAEs we found in the study included myelosuppression, hepatic insufficiency, pneumonitis, and endocrinopathies. The incidence of myelosuppression, hepatic insufficiency, and pneumonitis reported here is higher than previous studies.^21^, ^22^, ^23^ Approximately three‐quarter patients in our study received combination regimens, which contained ICI and other drugs. Chemotherapy is the most frequent strategy used in combination with ICIs, and may contribute to the high incidence rate. Additionally, real‐world settings include patients with a wider range, and results from these patients will be different from that of clinical trials with less population heterogeneity and better quality. Fortunately, most myelosuppression and hepatic insufficiency can be resolved after supportive therapy. However, it should be noted that more than 30% of patients who presented pneumonitis died from severe irAEs in our cohort. This highlights the need for regular follow‐up and timely detection of immune‐related pneumonitis. In fact, cytotoxic chemotherapy drugs such as docetaxel or gemcitabine, which are often used as a combination of ICIs, can also contribute to pneumonitis.^24^, ^25^ Pneumonitis is defined as the inflammation of the lung parenchyma and is usually identified by chest radiography.^26^ When symptoms occur and/or abnormal imaging manifestations are identified, clinicians should carefully rule out the possibilities of infectious etiologies or malignant infiltration, and guide patients for systematic management. Similar to skin toxicities, the endocrine toxicities may present many manifestations including thyroid dysfunction, adrenal insufficiency, and type 1 diabetes mellitus. For patients who develop hyperthyroidism, monitoring thyroid function is necessary as some will transfer to hypothyroidism, according to the results of ours and others.^27^

Some irAEs are relatively rare but life‐threatening. In our cohort, some patients died from severe colitis, myocarditis, pulmonary embolism, hypophysitis, or encephalitis. Furthermore, although most irAEs occur early after ICI treatment, some irAEs will present in a delayed pattern, such as colitis, rash and pneumonitis.^11^, ^28^ Importantly, for a certain irAE, the onset time for each individual may be significantly different. As shown in our results, time of irAEs onset appears to be varied across a wide range in real world settings. These situations require clinicians to make individualized diagnosis and treatment for irAEs. When corresponding symptoms appear, clinicians should be alert and make timely decisions.

The relationships of irAEs onset and prognosis have been studied previously.^12^, ^13^, ^14^ In line with previous studies, we found that patients with irAEs onset would achieve better prognosis than patients without irAEs, in the aspect of PFS and OS. Furthermore, we demonstrated that onset of skin toxicities would indicate a better PFS, which is also consistent with other studies.^29^, ^30^, ^31^ We also found that patients who presented myelosuppression during ICI treatment will achieve better PFS. Whether irAEs can serve as a robust predictor of efficacy needs to be further investigated.

Our study has some limitations. Firstly, as this is a single‐center study, extrapolation of the results is limited. Secondly, subgroup analysis based on specific ICI drugs, cancer types, and treatment combinations are not included in the study. However, the toxicity profile and incidence of irAEs varies among ICI drugs has been reported.^32^ In addition, cytotoxic T‐lymphocyte‐associated antigen 4 (CTLA‐4) antibodies were not used in our center and the corresponding data cannot be acquired. Thirdly, OS data should be interpreted with caution as fewer than 50% patients had OS event at the study cutoff date, albeit the sensitivity analysis reflects a similar trend in our investigation of the correlation of OS and irAEs onset. What is more, a longer follow‐up time will be needed for some rare and delayed irAEs, such as primary adrenal insufficiency which is not observed in our cohort currently. As a real‐world observational study with a pan‐cancer cohort, a limitation of mixing multiple treatment regimens may inevitably exist. In the future, studies with a larger sample size are needed to perform subgroup analysis for specific combinations.

In conclusion, irAEs are commonly observed in pan‐cancer patients treated with ICIs in real‐world settings. Skin toxicities are the most common and the earliest irAEs presented. Most irAEs occur early after ICIs treatment and are mild and manageable. However, irAEs are still needed to be carefully monitored as some will present at later time or can be life‐threatening, especially pneumonitis as we observed. Patients with any irAE onset may achieve a better prognosis than those without irAEs, and presentation of skin or blood toxicities will indicate a better PFS. Further real‐world studies with larger sample size and longer follow‐up time are needed to better understand irAEs that are relatively rare or present with a delayed pattern.

## AUTHOR CONTRIBUTIONS

**Xiaoxiao Ge:** Conceptualization (equal); data curation (equal); formal analysis (equal); validation (equal); writing – original draft (equal); writing – review and editing (equal). **Weiping Jiang:** Data curation (equal); formal analysis (equal); validation (equal). **Hongqing Li:** Data curation (equal); formal analysis (equal); validation (equal). **Yanxu Wu:** Data curation (equal); formal analysis (equal); validation (equal). **Xiangyang Li:** Data curation (equal); formal analysis (equal); methodology (lead); supervision (lead); validation (equal). **Shaohua Cui:** Conceptualization (lead); data curation (equal); methodology (equal); project administration (lead); supervision (lead); writing – original draft (equal); writing – review and editing (equal).

## FUNDING INFORMATION

This work was supported by the Key Discipline Project of Huadong Hospital, Fudan University (grant number: ZDXK2216), and the Key Project of Huadong Hospital, Fudan University (grant number: 2019jc028). The funders had no role in the planning or execution of the study.

## CONFLICT OF INTEREST STATEMENT

The authors have no conflict of interest.

## ETHICS STATEMENT

This study was approved by the Ethics Committee of Huadong Hospital, Fudan University, Shanghai (Ethical approval number: 20220100).

## Supporting information


Table S1.

## Data Availability

Data available on request from the authors.
