# Supplementary material for: Immune‐related adverse events and outcomes among pan‐cancer patients receiving immune checkpoint inhibitors: A monocentric real‐world observational study
Source: Cancer Med. 2023 Aug 11;12(18):18491–502. doi: 10.1002/cam4.6449 (PMC10557884; doi:10.1002/cam4.6449)
Supplement: Supplementary file 1 — Table S1. [file CAM4-12-18491-s001.docx]

**Supplementary Table 1.** Specific ICIs used for the 241 patients.

| **ICI type** | **ICI drug** | **n** | **%** |
| --- | --- | --- | --- |
| PD-1 antibodies | Camrelizumab | 97 | 40.2 |
|  | Pembrolizumab | 58 | 24.1 |
|  | Sintilimab | 53 | 22.0 |
|  | Nivolumab | 11 | 4.6 |
|  | Toripalimab | 10 | 4.1 |
| PD-L1 antibodies | Atezolizumab | 6 | 2.5 |
|  | Durvalumab | 6 | 2.5 |

Abbreviations: ICI, immune checkpoint inhibitor; PD-1, programmed cell death 1; PD-L1, programmed cell death ligand 1.
